# Prevalence of kidney stones in mainland China: A systematic review

**DOI:** 10.1038/srep41630

**Published:** 2017-01-31

**Authors:** Wenying Wang, Jingyuan Fan, Guifeng Huang, Jun Li, Xi Zhu, Ye Tian, Li Su

**Affiliations:** 1 Department of Urology, Beijing Friendship Hospital, Capital Medical University, Beijing, 100050, China; 2 School of Public Health of Guangxi Medical University, Guangxi Colleges and Universities Key Laboratory of the Prevention and Control of High Prevalence Diseases, Nanning, Guangxi, 530021, China

## Abstract

The data on the prevalence of kidney stones in mainland China are still lacking. We performed the present meta-analysis to assess the stone prevalence in mainland China from 1990 through 2016. A total of 18 articles were included. The pooled overall prevalence was 7.54% (95% CI, 5.94–9.15). The prevalence in age groups of <20 years, 20–29 years, 30–39 years, 40–49 years, 50–59 years, and 60 years and older was 0.27%, 3.15%, 5.96%, 8.18%, 9.14%, and 9.68%, respectively, showing that it increased with age. Moreover, the prevalence was 10.34% in males and 6.62% in females, with an odds ratio (OR) of 1.63 [95% CI: 1.51–1.76], indicating that males are more likely to suffer from this disease than females. However, urban areas (6.03%, 95% CI: 3.39–8.68) and rural areas (7.48%, 95% CI: 3.39–11.57) did not differ in the stone prevalence rate (OR = 0.84, 95% CI: 0.42–1.68). The prevalence in the year groups of 1991–2000, 2001–2010, and 2011 to date was 5.95%, 8.86%, and 10.63%, respectively, which indicated an increasing trend. Further high-quality surveys throughout mainland China are needed to confirm these findings.

Kidney stones (nephrolithiasis) are a common urological disease, which can seriously affect health and quality of life in populations worldwide. They can cause significant morbidity including urinary tract infection, flank pain, hydronephrosis, decreased renal function, etc. Renal colic caused by a stone is the common presentation to the emergency department, and is a frequent task of the emergency physician and a significant burden for healthcare systems^1^. Due to a high recurrence rate of up to 50% within the subsequent 5–10 years after the first episode^2^, the rate of repeated operative intervention is high. For patients with cystine urolithiasis, the mean interventional rate will reach 10.6 procedures per patient, and due to lack of satisfactory therapeutic options, these patients will have a lower quality of life compared with the general public^3^. Because the prime workforce (ages 20–60 yr) is at high risk of this disease, it may also lead to a huge burden on both families and society^4^. In general, kidney stone disease has recently gained considerable global visibility; it is a public health problem with a significant impact on people’s health that cannot be ignored. Moreover, the financial burden caused by nephrolithiasis is huge; it accounted for 2.1 billion dollars in the US in 2000^5^.

It was reported, however, that the prevalence of nephrolithiasis varies in different countries across the world. A previous study by Romero *et al*. reported that the rate in Western countries ranged from 0.1% to 14.8%^6^. However, in Asia, Taiwan was likely to have a 9.6% of population at risk of developing upper urinary calculi^7^. Moreover, according to the previous studies, the incidence of nephrolithiasis has been reported to be increasing globally^6^,^8^, even having appeared to nearly double in the last 15 years (1988–1994: 6.3%; 2007–2010: 10.3%) among men in the US^9^,^10^. In addition, although lower than that in adults, its rising trend in children is also a cause for concern. Evidence showed that the incidence of pediatric nephrolithiasis increased at a rate of 4% per year^11^. In China, because of the melamine-contamination event in 2008, it was estimated that approximately 300,000 children were diagnosed with nephrolithiasis^12^. The pathogenesis is still elusive, but usually stone formation is influenced by genetic and environmental factors. It is known that nephrolithiasis is strongly related to the loss of kidney function^13^, and it has now been recognized as a systemic disorder associated with increased risk of several common diseases such as bone loss, coronary artery disease, hypertension, type 2 diabetes mellitus, and metabolic syndrome^14^.

China has the largest population in the world; thus, the number of kidney stone patients in China could be enormous. It is known that the nationwide epidemiology of nephrolithiasis plays an essential role in assessing the disease burden and developing relevant policies, but the prevalence in survey studies varied substantially from 1.61%^15^ to 20.45%^16^. Many factors such as geographical province, survey date, and study population could easily influence the results; therefore, the data estimated by a single study were inappropriate to represent the national prevalence. Moreover, multicenter studies were also insufficient to generalize the prevalence. Therefore, to calculate the stone prevalence in the population of mainland China, the results conducted by meta-analysis might be appropriate.

To the best of our knowledge, this is the first meta-analysis based on epidemiological surveys to determine the national prevalence rate and the characteristics of nephrolithiasis in mainland China. In addition, to investigate the epidemiological characteristics for the prevention of this disease, prevalence according to gender, age, year, location, and some other risk factors was calculated in our meta-analysis.

## Results

### Study identification

**Figure 1:**
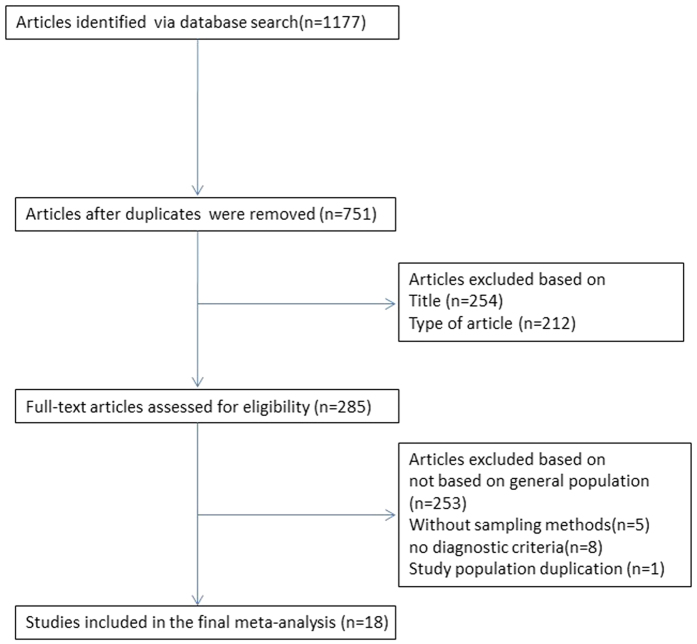
Flow chart of the article selection process to evaluate the prevalence of kidney stones in mainland China.

We identified 1177 articles in a database search, 100 in English and 1077 in Chinese. The search results were as follows: PubMed (n = 100), CNKI (n = 659), the WanFang database (n = 199), the Chongqing VIP database (n = 71), and CBM (n = 148). After the titles and abstracts were read, 892 studies were subsequently removed (because of duplications between databases or being unrelated to our study) and, subsequently, 285 articles were retrieved for full-text reading. Among these, 267 articles met the exclusion criteria, of which 253 were not based on a general population, 8 did not provide diagnostic criteria, 5 had no sampling methods, and 1 was a duplicate publication. Finally, 18 articles were included in our meta-analysis^15^,^16^,^17^,^18^,^19^,^20^,^21^,^22^,^23^,^24^,^25^,^26^,^27^,^28^,^29^,^30^,^31^,^32^ (Fig. 1).

### Characteristics of included studies and quality assessment

All 18 articles were cross-sectional studies, published in Chinese, conducted in eight provinces in mainland China (Beijing, Guangdong, Guangxi, Sichuan, Shanghai, Zhejiang, Xinjiang, and Hebei) from 1990 to 2016. On the basis of the ultrasound examination, 7032 patients with kidney stones were identified in a total of 115,087 individuals in the 18 included studies. The prevalence ranged from 1.61% to 20.45% in this group. The quality assessment was performed using the Strengthening the Reporting of Observational Studies in Epidemiology (STROBE) guideline. Among the 18 eligible studies, 2 studies scored 10, 9 studies scored 9, 5 studies scored 8, 1 study scored 7, and 1 study scored 6. More details can be seen in Table 1 and Supplementary Table 1.

### The epidemiology of kidney stones

#### Overall prevalence

**Figure 2:**
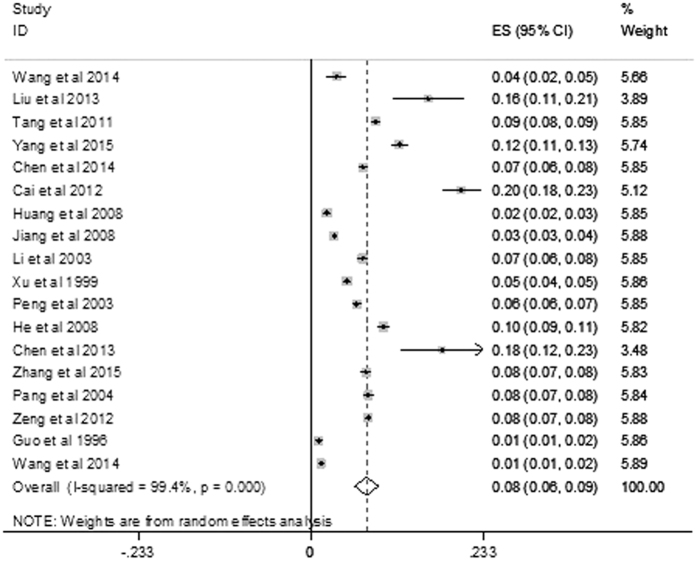
Forest plot for the overall estimate of the prevalence of kidney stones.

The prevalence of kidney stones varied from 1.61%^15^ to 20.45%^16^; the pooled overall prevalence was 7.54% (95% CI: 5.94–9.15) (Fig. 2).

#### Prevalence by gender

Fourteen studies showed the prevalence by gender subgroup, estimating a rate of 10.34% (95% CI: 7.96–12.72) in males and 6.62% (95% CI: 5.12–8.12) in females. Compared with females, males seemed to have a higher risk for suffering from kidney stone disease (OR = 1.63, 95% CI: 1.51–1.76) (Supplementary Fig. 1).

#### Prevalence by province and location

The highest prevalence was 13.69% in Sichuan, whereas the lowest was 1.36% in Hebei province. The prevalence in urban and rural areas was 6.03% (95% CI, 3.39–8.68) and 7.48% (95% CI, 3.39–11.57), respectively (Table 2). However, urban and rural areas did not differ in stone prevalence, with an OR of 0.84 (95% CI: 0.42–1.68) (Supplementary Fig. 2).

#### Prevalence by age

**Figure 3:**
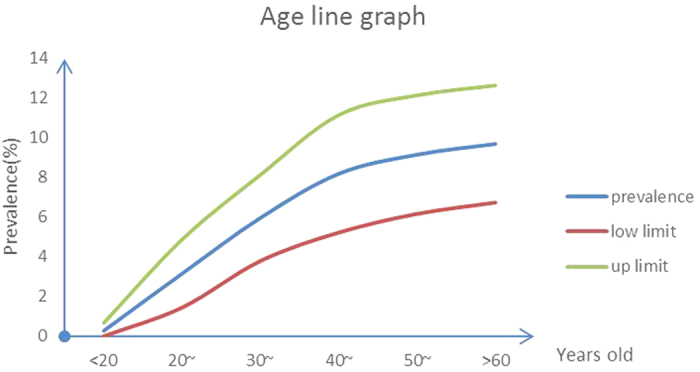
Prevalence of kidney stones by different age groups.

Several studies evaluated the age-specific prevalence, and the age groups adopted were as follows: <20 years, 20–29 years, 30–39 years, 40–49 years, 50–59 years, and 60 years or older. The rate in these age groups was 0.27%, 3.15%, 5.96%, 8.18%, 9.14%, and 9.68%, respectively (Table 2). Thus, it can be easily concluded that the prevalence increased with age (Fig. 3).

#### Prevalence by year

**Figure 4:**
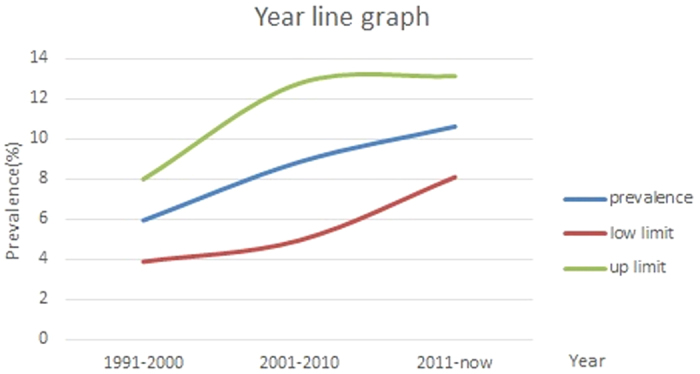
Pooled estimated prevalence of kidney stones in mainland China with corresponding 95% confidence intervals from different survey periods.

Prevalence in the year groups of 1991–2000, 2001–2010, and 2011 to date was 5.95%, 8.86%, and 10.63%, respectively, which showed an increasing trend (Fig. 4). Besides, compared to the 1990s, the prevalence of nephrolithiasis nearly doubled in recent years, which is worth attention and requires more surveys for support.

#### Prevalence by urinary infection

In addition, people with urinary infection (31.99%, 95% CI: 11.35–52.62) appeared to have a significantly higher risk of kidney stones than those without urinary infection (12.52%, 95% CI: 9.03–16.02) (OR = 3.01, 95% CI: 1.79–5.07) (Supplementary Fig. 3).

#### Prevalence by education level

Only four studies that evaluated the prevalence by education level were entered into this analysis, and the results were as follows: elementary and below (9.84%, 95% CI: 7.40–12.29), junior high (8.12%, 95% CI: 4.76–11.48), senior high (7.11%, 95% CI: 3.92–10.31), and college or above (7.91%, 95% CI: 3.31–12.51) (Table 2). Note that the higher the education level, the lower might be the frequency of stones. However, this potential tendency is subject to many confounding factors such as socioeconomic status as well as region, and requires more support.

#### Sources of heterogeneity, sensitivity analysis, and publication bias

Heterogeneity between studies was significant, and was estimated at I^2^ = 99.4% in this meta-analysis. Therefore, the random-effects model was used in the meta-analysis. The sources of heterogeneity by univariate meta-regression were conducted with several assessed covariates, including year of survey, province, age distribution, sampling methods, and quality score of bias risk. The results showed that the published year and risk of bias may be the sources of heterogeneity. With regard to “leave one out” sensitivity analysis, we found that the pooled prevalence became higher when the study by Wang *et al*.^29^ was removed; however, the pooled prevalence was lower when the study by Zeng *et al*.^25^ was removed. No publication bias was found in the present study according to the results of both Begg’s test and Egger’s test (p > 0.05), although the funnel plots were asymmetrical.

## Discussion

To our knowledge, this is the first meta-analysis estimating the overall prevalence of kidney stones in an adult population in mainland China. In our meta-analysis, the pooled overall prevalence was 7.54%. The prevalence increased with age, and gender was also a principal determinant of the prevalence (10.34% male, 6.62% female). Several factors were found to significantly contribute to disease development, such as location (urban/rural), urinary infection history, and education level.

Kidney stone disease is common in industrialized countries. Patients with this disease suffer agonizing pain and vast economic impact. However, the prevalence of stone disease in the Chinese population remains unclear. Previously, several attempts had been made to indicate the stone prevalence at regional and national levels. In comparison to these results, our estimate (7.54%) is based on the evidence of epidemiological surveys. It is a little higher than the report by Zeng *et al*. in 2013 in a multicenter cross-sectional survey, which indicated that the prevalence of urinary stones (including kidney, bladder, and ureter stones) was 6.5%^33^. This difference might be real or due to the relatively fewer male subjects in Zeng’s study (3792 males vs. 5518 females). Another large-sample health examination survey including 1,169,651 urban inhabitants showed that the overall prevalence was 4.0% in 2008^34^, but the data from healthy examination subjects possibly lack the representation of a nonrandom sample population, which might cause selection bias and underestimation of the prevalence.

### Growing number of patients

To our knowledge, the first report about kidney stone prevalence among the Chinese population was in 1977, in Guangdong province, and the estimate was 1.16%^35^. On the basis of the included studies in our meta-analysis, the prevalence was estimated at 7.70% in Guangdong and 7.54% nationwide, indicating that the prevalence increased by more than 5 times in the past few decades. This increase was also found in other countries around the world. Prevalence in the United States has increased from 3.8% in the 1970 s to 5.2% in the 1990 s and 8.8% in the 2000s^9^,^10^. This phenomenon was also found in surveys from Germany, where the stone prevalence increased from 4% to 4.7% from 1997 to 2001, and the prevalence among those older than 65 years increased from 6.8% to 9.5%^36^. The present meta-analysis showed that the prevalence in the year groups of 1991–2000, 2001–2010, and 2011 to date was 5.95%, 8.86%, and 10.63%, respectively, which indicated an increasing trend. One explanation for this increase is the increasing positive rate of asymptomatic stones by the growing use of and developing sensitivity to imaging devices. The other explanation is that China has experienced great social development and rapid economic changes in the past few decades, and has adopted a more Western-like lifestyle, which may contribute to the rising burden of stone disease. Thus, the prevalence is expected to increase in the following decades.

### Increasing risk along with age

The overall improvement in healthcare has led to an increased number of aged people. Unprecedented attention is being paid to age-related diseases. Similar to former studies^34^, the stone prevalence was found to increase with age in this meta-analysis. Compared with the age group of 20–30 years (3.15%), the prevalence nearly tripled in the age group of 60 and older (9.68%). The pathophysiological explanation of the age trend was complicated, and the preventive value was limited. On the one hand, several systemic disorders increased risk of stone formation, such as diabetes mellitus, hypertension, and obesity, which are more common among the elder population^37^. On the other hand, dietary factors such as a high intake of animal protein, sodium, and sucrose could increase calcium excretion independent of calcium intake, which increases the risk of stone formation^38^. Correlated to this, the intake of condiments is higher because of the degradation of taste in elder people. Moreover, higher incidence of urinary infections in the elder population also increases the risk of nephrolithiasis^39^. Further surveys to assess the risk factors among the elder population are warranted.

### Narrowing the gap with developed countries

Previous studies indicated that the overall probability of stone disease differed in various parts of world before 2000, which was 1% to 5% in Asia, 5% to 9% in Europe, 13% in North America, and 20% in Saudi Arabia^40^. This gap is narrowing quickly, although the prevalence in developed countries doubled or even tripled during the last quarter of the 19th century. Although there are various reasons for this, the stone formation was reported to have a strong relationship with obesity and dietary components^41^. High obesity incidence and high animal protein intake were previously considered risk factors only in Westerners; currently, these risk factors are becoming increasingly common in the Chinese population^42^. Moreover, studies showed that both the improvement in diagnostic procedures and environmental changes in developing countries contributed significantly to this modification^6^.

### Existing gender differences

Historically, nephrolithiasis was seen more commonly in males than in females. However, recent evidence suggests the gender ratio has changed; the overall man-to-woman ratio of incidence decreased from 3.1 to 1.3^43^. Besides, a study based on the Nationwide Inpatient Sample indicated that between 1997 and 2002 this ratio decreased from 1.7 to 1.3^44^. Our meta-analysis estimated the prevalence of males and females to be 10.34% and 6.62%, respectively (OR = 1.63). Compared with the gender ratio of kidney stones in patients in the 1950 s (380/139), the gender gap still exists but has reduced significantly. This change may result from a variety of etiologies. First, obesity/overweight, associated with an increased risk of nephrolithiasis^45^, has recently been found to be more common in females than in males^46^. Second, dietary habits such as high intake of animal protein, high salt intake, and low calcium intake, which are associated with stone formation, were previously more common in males. The same dietary habits are now common in females and have contributed to this change as well^47^. Third, a stressful life event was considered to be related to the incidence of symptomatic kidney stones^48^. In Chinese traditional society, we believed that a good girl should not be highly knowledgeable, and that women should stay at home rather than work outside the home. The social progress made by women in the past few decades has made their lives more stressful. Women suffer from more occupational stress than do men in some cases.

### Consideration of regional distribution

The overall prevalence of kidney stone disease varies in different parts of the world. Similarly, it differed in provinces of China, and this difference has not yet been highlighted. In this meta-analysis, regional prevalence varied from 1.36% to 13.69%; the lowest prevalence was observed in Hebei, and the highest was in Sichuan. As shown in previous studies, climatologic factors most likely responsible for the prevalence are heat, more concentrated urine, sunlight, diet, water hardness, fluid intake, and comorbid conditions such as obesity and hypertension. According to this finding, the high prevalence in Sichuan and Zhejiang may due to the preference of tea. Heat and sunlight may contribute to the high incidence rate in Guangdong, Guangxi, and Xinjiang. Nevertheless, the studies could not adequately explain the geographic variation in stone prevalence in mainland China. With regard to the difference between urban and rural areas (6.03% vs. 7.48%), the lack of access to water or bathroom facilities may lead to lower fluid intake and, thus, a higher risk of stone formation^38^. More detailed evidence such as climate, diet, and other environmental factors is needed to better understand the potential causes of the regional variation in the prevalence of kidney stones.

Some other characteristics of nephrolithiasis in the mainland still remain unclear, including stone composition, treatment development, etc. Modification diet, body habitus, and management of urinary tract infection will have some effect on the proportion of stone composition. A decreased trend in the proportion of struvite stones was observed in Australia, which may reflect the improvement of urinary tract infections within the Australian population^49^. In mainland China, further studies are needed to investigate the changes of stone composition to prevent and reduce the risk of subsequent stone formation. The management of nephrolithiasis has changed a great deal over the past decades; considerable increase in flexible ureteroscopy and decrease in extra-corporeal shockwave lithotripsy and percutaneous nephrolithotomy were observed in Australia^50^, UK^51^, and other countries^52^. In mainland China, less invasive techniques have been widely used; flexible ureteroscopy procedures were not only performed in adults, but also in pediatric patients^53^.

Considering the heterogeneity of this meta-analysis, some factors including gender, age, location (urban/rural), urinary infection history, and education level contribute to the heterogeneity. To mitigate heterogeneity, we performed subgroup and sensitivity analyses; we found that the heterogeneity was still very high within the subgroups. Some other factors such as climate, diet, and environmental factors also cause heterogeneity; unfortunately, we could not obtain these data from the component studies. This meta-analysis also has some limitations. First, because the epidemiological studies were not performed in all the provinces, the data could be obtained only from eight provinces in mainland China. Second, the rapid economic development and increase in radiological imaging in mainland China will contribute to an increased diagnosis of asymptomatic kidney stones; hence, the results of this meta-analysis should be interpreted with care. Nevertheless, we conducted this meta-analysis on the basis of sufficient data and generated a precise estimate of the prevalence of nephrolithiasis.

In summary, nephrolithiasis is prevalent in mainland China, and the prevalence increases with age. Males are more vulnerable to suffer from this disease than females. An increasing trend with decade was observed. Further high-quality surveys aiming to assess the risk factors for kidney stones are warranted. In our opinion, the results of this study deserve the attention of policymakers and support the planning and implementation of public health policies.

## Methods

### Literature Search

The present meta-analysis was performed following the guidelines in the Preferred Reporting Items for Systematic Reviews and Meta-Analysis (PRISMA) statement^54^. Data for this meta-analysis were comprehensively identified by searching the Chinese National Knowledge Infrastructure database (CNKI), the WanFang database, the Chongqing VIP database, the Chinese Biological Medical Literature database (CBM), PubMed, and Embase (January, 1990 to February, 2016) with the following search terms: “kidney stones,” “renal calculus,” “urinary stone,” “urolithiasis,” “nephrolithiasis,” “nephrocalcinosis,” “prevalence,” “epidemiology,” and “China.” We reviewed only those papers published in either Chinese or English. In addition, we performed a further search by perusing the references of review articles and e-mailing the authors requesting full-text articles to include all available data in this meta-analysis.

### Inclusion and exclusion criteria

Articles were considered eligible for inclusion if they met the following criteria: (1) cross-sectional studies; (2) conducted in mainland China (not including Hong Kong, Taiwan, and Macao); (3) community-based or population-based studies; (4) evaluated the prevalence of kidney stones; (5) published in Chinese and/or English; and (6) used appropriate diagnostic criteria. If several articles were based on the same or approximate populations, the study with the better/best epidemiological quality (e.g., the larger sample sizes, more normative diagnostic methodology, and/or more systematic statistical analysis) was included.

Articles were excluded if: (1) the survey was not a random sampling; (2) the study was based on specific populations; (3) study population duplication; (4) review articles; and (5) the prevalence was estimated by questionnaire or self-report only and no diagnostic criteria.

### Data extraction

Two authors independently extracted the available data from the included studies, and the disagreement was resolved through discussion. The extracted data included the first author, year of publication, type of survey (community/population based), age range and age group, sampling methods, province where the survey was conducted, location (urban/rural), diagnostic criteria, sample size, the response rate, the prevalence, and sex distribution. Factors that influenced the prevalence were also extracted.

### Quality assessment

In this meta-analysis, the quality assessment was independently conducted by two authors using the Strengthening the Reporting of Observational Studies in Epidemiology (STROBE) guideline^55^, which is composed of five items, and the risk of bias of each item is divided into three levels (low risk = 0, moderate risk = 1, and high risk = 2). The total score represented the quality score of risk of bias of individual studies (Supplementary Table 2).

### Statistical analysis

In this meta-analysis, the pooled prevalence was calculated using STATA 11.1 (Stata Corporation, College Station, Texas, USA). For the comparison in groups of gender, infection history, and location, the odds ratio (OR) pictures were calculated by the Review Manager (RevMan) version 5.1. Moreover, heterogeneity between studies was assessed with Cochran’s Q statistic and the I^2^ index. If I^2^ ≧ 50% or p < 0.05, the heterogeneity was considered to be significant. To determine the sources of heterogeneity, we conducted a meta-regression of the year of study, province, age range, sample size, and scores in the risk of bias. Sensitivity analysis was performed to detect the effects of one single study on the pooled results when excluding the included studies one at a time. Finally, publication bias was analyzed by Egger’s test and Begg’s test, and we considered p < 0.05 to be significant.

## Additional Information

**How to cite this article**: Wang, W. *et al*. Prevalence of kidney stones in mainland China: A systematic review. *Sci. Rep.* **7**, 41630; doi: 10.1038/srep41630 (2017).

**Publisher's note:** Springer Nature remains neutral with regard to jurisdictional claims in published maps and institutional affiliations.

## Supplementary Material

Supplementary Information

## Figures and Tables

**Table 1:** Characteristics of the included studies and quality scores for assessing the risk of bias in the individual studies.

Study	Year	Age	Sample methods	U&R	Province	Sample size (N)	Cases (n)	Prevalence (%)	Quality score
Guo *et al*.^15^	NA	4–87	random	R	Hebei	1487	24	1.61	8
Xu *et al*.^17^	1997	>15	stratified cluster random	U	Guangdong	7399	360	4.87	9
Li *et al*.^18^	2000	>15	stratified cluster random	U&R	Guangdong	7625	535	7.02	8
Peng *et al*.^19^	NA	>15	stratified cluster random	U	Guangdong	6224	384	6.17	8
Pang *et al*.^20^	2002	> = 20	cluster stratified random	U&R	Guangxi	7259	568	7.82	8
Jiang *et al*.^23^	2006	> = 16	cluster stratified random	U&R	Shanghai	12565	396	3.15	9
He *et al*.^21^	2006–2007	>15	stratified cluster random	U	Zhejiang	5915	578	9.77	9
Huang *et al*.^22^	NA	> = 18	mutistage cluster random	U	Shanghai	2554	56	2.19	9
Tang *et al*.^24^	NA	> = 18	stratified multi-stage random	U&R	Guangxi	8957	784	8.75	10
Cai *et al*.^16^	2011	> = 10	stratified random	R	Sichuan	939	192	20.45	8
Zeng *et al*.^25^	2012	>10	cluster stratified random	U	Guangdong	19000	1482	7.80	9
Chen *et al*.^26^	2009	> = 6	random	R	Guangdong	185	33	17.84	7
Liu *et al*.^27^	2011	NA	cluster random	R	Guangdong	227	36	15.86	6
Wang *et al*.^30^	2012	> = 18	stratified system random	U	Beijing	715	25	3.50	9
Chen *et al*.^28^	NA	26–75	steatified random	R	Sichuan	6815	482	7.07	9
Wang *et al*.^29^	NA	>15	cluster stratified random	U&R	Hebei	17854	1752	9.81	10
Yang *et al*. 2015	2012	> = 18	cluster mutistage random	R	Guangxi	3475	418	12.03	9
Zhang *et al*.^32^	2012–2013	>15	cluster random	U&R	Xinjiang	5892	440	7.47	9

U: urban; R: rural.

**Table 2:** Prevalence of kidney stones in mainland China and subgroup analysis.

Variable	Kidney stone						
	Number of surveys	Sample size	Cases	Prevalence (%)	95% CI	I^2^	
Overall		18	115087	7032	7.54	5.94 to 9.15	99.4
Year	1991–2000	2	15024	895	5.95	3.89 to 8.00	96.6
	2001–2010	4	25924	1575	8.86	4.95 to 12.78	99.2
	2011-now	6	30248	2593	10.63	8.11 to 13.14	97.5
	NA	6	43891	1969	4.46	1.86 to 7.06	99.4
Age	<20	6	1444	14	0.27	−0.13 to 0.67	59.5
	20~	8	6855	249	3.15	1.44 to 4.87	96.9
	30~	8	8902	453	5.96	3.79 to 8.13	95.2
	40~	8	8216	511	8.18	5.22 to 11.14	96.5
	50~	8	8641	626	9.14	6.16 to 12.13	95.1
	>60	8	8623	696	9.68	6.73 to 12.63	94.8
gender	male	14	49080	3672	10.34	7.96 to 12.72	99.1
	female	14	52294	2478	6.62	5.12 to 8.12	98.7
Province	Beijing	1	818	25	3.10	1.91 to 4.29	NA
	Guangdong	6	40660	2830	7.70	6.25 to 9.15	95.7
	Guangxi	3	19691	1770	9.48	7.53 to 11.43	95.4
	Sichuan	2	7754	674	13.69	0.66 to 26.72	99.0
	Shanghai	2	15119	452	2.73	1.75 to 3.71	89.1
	Zhejiang	1	5915	578	9.80	9.04 to 10.56	NA
	Xinjiang	1	5892	440	7.50	6.83 to 8.17	NA
	Hebei	2	19341	263	1.36	1.16 to 1.56	10.1
Location	Urban	4	18934	1082	6.03	3.39 to 8.68	98.5
	Rural	4	15748	1106	7.48	3.39 to 11.57	99.1
Infection history	yes	3	716	155	31.99	11.35 to 52.62	94.1
	no	3	9614	1032	12.52	9.03 to 16.02	95.9
Educational level	elemantary and below	4	9622	920	9.84	7.40 to 12.29	94.2
	junior high	4	7686	589	8.12	4.76 to 11.48	97.0
	senior high	4	4692	241	7.11	3.92 to 10.31	95.3
	college and above	4	2250	135	7.91	3.31 to 12.51	94.9
Risk of bias	≥9	11	91141	5267	6.19	4.19 to 8.20	99.5
	<9	5	23946	1765	10.11	7.14 to 13.09	98.8
