# Supplementary material for: Prevalence of kidney stones in mainland China: A systematic review
Source: Sci Rep. 2017 Jan 31;7:41630. doi: 10.1038/srep41630 (PMC5282506; doi:10.1038/srep41630)

# Prevalence of kidney stones in mainland China: a systematic review

Wenying Wang, Jingyuan Fan, Guifeng Huang, Jun Li, Xi Zhu, Ye Tian and Li Su

Correspondence should be addressed to L.S.(E-mail: bjminiaowwy@sina.com)

**Supplementary Table 1.** Quality scores for assessing the risk of bias in the individual studies

**Supplementary Table 2**. Tool for assessing the risk of bias

**Supplementary Fig 1**. The odds ratio of prevalence by gender (males vs. females)

**Supplementary Fig 2**. The odds ratio of prevalence by location (urban vs. rural areas)

**Supplementary Fig 3**. The odds ratio of prevalence by urinary infection (with vs. without infection)

| **Supplementary Table1**. Quality scores for assessing the risk of bias in the individual studies | | | | | | |  | | |  | |
| --- | --- | --- | --- | --- | --- | --- | --- | --- | --- | --- | --- |
| study | case in total | | | Quality score | | | | | | | |
|  | N | n | prevalence(%) | Total score | sample population | sample size | | participation | outcome assessment | | analytical methods |
| Wang et al 2014 | 715 | 25 | 3.50 | 9 | 2 | 1 | | 2 | 2 | | 2 |
| Liu et al 2013 | 227 | 36 | 15.86 | 6 | 1 | 1 | | 0 | 2 | | 2 |
| Tang et al 2011 | 8957 | 784 | 8.75 | 10 | 2 | 2 | | 2 | 2 | | 2 |
| Yang et al 2015 | 3475 | 418 | 12.03 | 9 | 2 | 1 | | 2 | 2 | | 2 |
| Chen et al 2014 | 6815 | 482 | 7.07 | 9 | 2 | 1 | | 2 | 2 | | 2 |
| Cai et al 2012 | 939 | 192 | 20.45 | 8 | 1 | 1 | | 2 | 2 | | 2 |
| Huang et al 2008 | 2554 | 56 | 2.19 | 9 | 2 | 1 | | 2 | 2 | | 2 |
| Jiang et al 2008 | 12565 | 396 | 3.15 | 9 | 2 | 2 | | 1 | 2 | | 2 |
| Li et al 2003 | 7625 | 535 | 7.02 | 8 | 2 | 1 | | 1 | 2 | | 2 |
| Xu et al 1999 | 7399 | 360 | 4.87 | 9 | 2 | 2 | | 1 | 2 | | 2 |
| Peng et al 2003 | 6224 | 384 | 6.17 | 8 | 2 | 1 | | 1 | 2 | | 2 |
| He et al 2008 | 5915 | 578 | 9.77 | 9 | 2 | 1 | | 2 | 2 | | 2 |
| Chen et al 2013 | 185 | 33 | 17.84 | 7 | 1 | 1 | | 1 | 2 | | 2 |
| Zhang et al 2015 | 5892 | 440 | 7.47 | 9 | 2 | 1 | | 2 | 2 | | 2 |
| Pang et al 2004 | 7259 | 568 | 7.82 | 8 | 2 | 1 | | 1 | 2 | | 2 |
| Zeng et al 2012 | 19000 | 1482 | 7.80 | 9 | 2 | 1 | | 2 | 2 | | 2 |
| Guo et al 1996 | 1487 | 17 | 1.11 | 8 | 1 | 1 | | 2 | 2 | | 2 |
| Wang et al 2014 | 17854 | 246 | 1.14 | 10 | 2 | 2 | | 2 | 2 | | 2 |

**Supplementary Table 2**. Tool for assessing the risk of bias

| Bias type | Low risk (score=2) | Moderate risk (score=1) | High risk (score=0) |
| --- | --- | --- | --- |
| Selection (sample population) | 1. Sample from general population not a select group;  2. Consecutive unselected population;  3. Rationale for case and control selection explained. | 1. Sample selected from large population but selection criteria not defined;  2. Sample selection ambiguous but may be representative;  3. Rationale for cases and controls not explained;  4. Eligibility criteria not explained;  5. Analysis to adjust for sampling strategy bias. | 1. Highly select population making it difficult to generalise finding;  2. Sample selection ambiguous and sample unlikely to be representative. |
| selection (sample size) | 1. sample size calculation performed and adequate. | 1. Sample size calculation performed and reasons for not meeting sample size given.  2. Sample size calculation not performed but all eligible persons studied; | 1. Sample size estimation unclear or only sub-sample studied. |
| Selection (participation rate) | 1. High response rate (>85%). | 1. Moderate response rate (70–85%). | 1. Low response rate (<70%). |
| Performance bias (outcome assessment) | 1. Diagnosis using consistent criteria and direct examination. | 1. Assessment from administrative database or register;  2. Assessment from hospital record or interviewer. | 1. Assessment from non-validated data or generic estimate from overall population. |
| Performance bias (analytical methods to control for bias) | 1. Analysis appropriate for type of sample (subgroup analysis/regression etc.). | 1. Analysis does not account for common adjustment. | 1. Data confusing. |

**Supplementary Fig 1**. The odds ratio of prevalence by gender (males vs females)


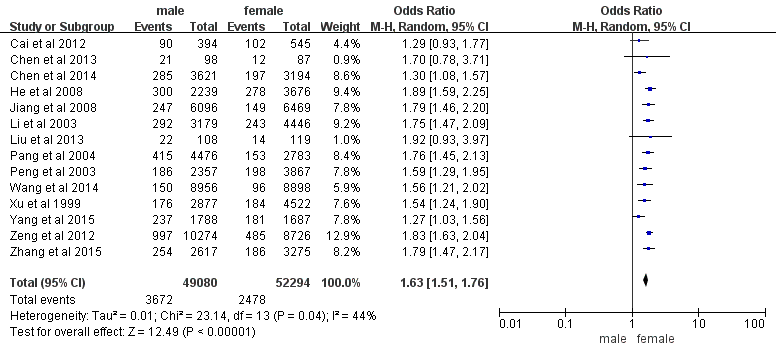


**Supplementary Fig 2**. The odds ratio of prevalence by location (urban vs rural areas)


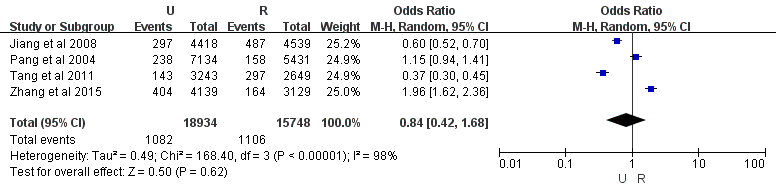


**Supplementary Fig 3**. The odds ratio of prevalence by urinary infection (with vs without infection)


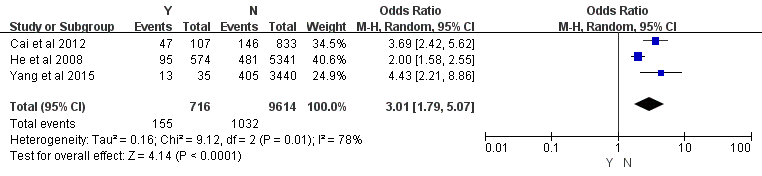

Supplement: Supplementary Information [file srep41630-s1.doc]
